# Sampling Sexual and Gender Minority Youth With UnACoRN (Understanding Affirming Communities, Relationships, and Networks): Lessons From a Web-Based Survey

**DOI:** 10.2196/44175

**Published:** 2023-01-12

**Authors:** Jorge Andrés Delgado-Ron, Thiyaana Jeyabalan, Sarah Watt, Stéphanie Black, Martha Gumprich, Travis Salway

**Affiliations:** 1 Reaffirm Collaborative Centre for Gender and Sexual Health Equity Vancouver, BC Canada; 2 Faculty of Health Sciences Simon Fraser University Burnaby, BC Canada; 3 Department of Population Medicine University of Guelph Guelph, ON Canada; 4 British Columbia Centre for Disease Control Vancouver, BC Canada

**Keywords:** sexual and gender minorities, transgender persons, sexual orientation, conversion therapy, web-based survey, surveys and questionnaires, adolescence, sexual minority youth, transgender youth, same-sex attraction, gender minority, health inequality, online recruitment, advertisement, social media recruitment

## Abstract

**Background:**

Periodic surveys of sexual and gender minority (SGM) populations are essential for monitoring and investigating health inequities. Recent legislative efforts to ban so-called conversion therapy make it necessary to adapt youth surveys to reach a wider range of SGM populations, including those <18 years of age and those who may not adopt an explicit two-spirit, lesbian, gay, bisexual, transgender, and queer (2S/LGBTQ) identity.

**Objective:**

We aimed to share our experiences in recruiting SGM youth through multiple in-person and online channels and to share lessons learned for future researchers.

**Methods:**

The Understanding Affirming Communities, Relationships, and Networks (UnACoRN) web-based survey collected anonymous data in English and French from 9679 mostly SGM respondents in the United States and Canada. Respondents were recruited from March 2022 to August 2022 using word-of-mouth referrals, leaflet distribution, bus advertisements, and paid and unpaid campaigns on social media and a pornography website. We analyzed the metadata provided by these and other online resources we used for recruitment (eg, Bitly and Qualtrics) and describe the campaign’s effectiveness by recruitment venue based on calculating the cost per completed survey and other secondary metrics.

**Results:**

Most participants were recruited through Meta (13,741/16,533, 83.1%), mainly through Instagram; 88.96% (visitors: 14,888/18,179) of our sample reached the survey through paid advertisements. Overall, the cost per survey was lower for Meta than Pornhub or the bus advertisements. Similarly, the proportion of visitors who started the survey was higher for Meta (8492/18,179, 46.7%) than Pornhub (58/18,179, 1.02%). Our subsample of 7037 residents of Canada had a similar geographic distribution to the general population, with an average absolute difference in proportion by province or territory of 1.4% compared to the Canadian census. Our US subsample included 2521 participants from all US states and the District of Columbia. A total of CAD $8571.58 (the currency exchange rate was US $1=CAD $1.25) was spent across 4 paid recruitment channels (Facebook, Instagram, PornHub, and bus advertisements). The most cost-effective tool of recruitment was Instagram, with an average cost per completed survey of CAD $1.48.

**Conclusions:**

UnACoRN recruited nearly 10,000 SGM youth in the United States and Canada, and the cost per survey was CAD $1.48. Researchers using online recruitment strategies should be aware of the differences in campaign management each website or social media platform offers and be prepared to engage with their framing (content selection and delivery) to correct any imbalances derived from it. Those who focus on SGM populations should consider how 2S/LGBTQ-oriented campaigns might deter participation from cisgender or heterosexual people or SGM people not identifying as 2S/LGBTQ, if relevant to their research design. Finally, those with limited resources may select fewer venues with lower cost per completed survey or that appeal more to their specific audience, if needed.

## Introduction

Periodic surveys of sexual and gender minority (SGM) populations have been an essential tool for monitoring and investigating health inequities, including those related to mental and sexual health [1]. The vast majority of these surveys use nonprobabilistic sampling [2]. The continued reliance on nonprobability sampling methods for research on SGM populations is explained by many factors, including their ability to generate a larger SGM sample size—on average—than probabilistic methods [3]; their methodological ease, relative to random, quasi-random, or respondent-driven sampling methods [4]; and their ability to sample from venues where the SGM target audience can be reached later on (eg, for purposes of health education or provision of resources). While national, general population probabilistic-sampling surveys in the United States and Canada (eg, the National Health and Nutrition Examination Survey and the Canadian Community Health Survey) have increasingly added SGM measures—thereby enabling the monitoring of some health outcomes across sexual orientations and gender modalities—SGM population–focused surveys allow researchers to ask in-depth questions regarding phenomena that uniquely affect SGM populations—domains that are not available from the broader general population surveys. Online recruitment, especially social media, has emerged as a cost-effective tool to access minority groups [5,6], including SGM youth in Australia [7], the United States [8,9], and Canada [10-12].

From 2019 to 2022, the Canadian federal government, along with several US states, debated and enacted legislation banning so-called conversion therapy [13,14]. Ongoing, in-depth data collection regarding experiences of conversion therapy is needed to evaluate the effectiveness of these bans. Because conversion therapy is poorly defined and closely related to a set of similar anti–two-spirit, lesbian, gay, bisexual, transgender, and queer (2S/LGBTQ) experiences known as sexual orientation and gender identity and expression change efforts (SOGIECE), measures of conversion therapy should be coupled with experiences of SOGIECE, heterosexism, cissexism, and related threats to health equity in SGM populations [15]. Moreover, because conversion therapy constitutes just one of a possible series of threats to 2S/LGBTQ identity expression, studies should also investigate the range of these threats (and opportunities, in the case of 2S/LGBTQ-affirming actions), including access to gender-affirming medical care and detransition experiences [16] and felt inclusion or exclusion in a wide range of social spheres (eg, team sports, religion, and school).

The Understanding Affirming Communities, Relationships, and Networks (UnACoRN) survey was a binational survey of young people (aged 15 to 29 years) living in Canada and the United States [17]. It predominantly included SGM participants, though cisgender-heterosexual (cis-het) people were also encouraged to participate (and were shown complementary questions related to attitudes toward 2S/LGBTQ people). Participants were primarily recruited from Canada, though some recruitment channels included participants from the United States, and these participants were included in some analyses (eg, those regarding rare phenomena). The overarching goal of the study was to understand the influence of personal values and beliefs and the role of families, communities, and other environments on sexual orientation, gender identity, and expression (SOGIE). We intend for our results to inform school administrators, lawmakers, and health care providers, as well as community organizations, religious organizations, sports teams, and similar institutions, about their potential role in fostering mental health and well-being, particularly among 2S/LGBTQ youth.

In this paper, we aimed to share our experiences in the recruitment of SGM youth through multiple in-person and online channels and to share lessons learned for future researchers. We hypothesized that online venues would be more cost-effective than in-person venues. We also hypothesized that combining in-person and online methods would help us recruit a sample that aligned with the census distribution of Canadian youth across provinces and territories.

## Methods

### Questionnaire

The UnACoRN survey was developed through an iterative process of consultation with experts in SGM population health and SGM community leaders that shared the research team’s awareness of the need to constantly monitor and address ongoing disparities that affect SGM populations. The survey content was prioritized as follows: first, we aimed to collect data on topics that were otherwise understudied in SGM population health; second, we prioritized topics related to conversion therapy and SOGIECE; and third, we emphasized theoretically or empirically relevant variables that may explain the elevated rates of adverse mental health and sexual health outcomes among SGM populations [18-20]. Validated scales were used for mental health outcomes (ie, the General Anxiety Disorder–7, Patient Health Questionnaire–9, and the Suicide Behaviors Questionnaire–Revised scales) and for explanatory variables when available (eg, the self-continuity scale) [21,22].

**Figure 1:**
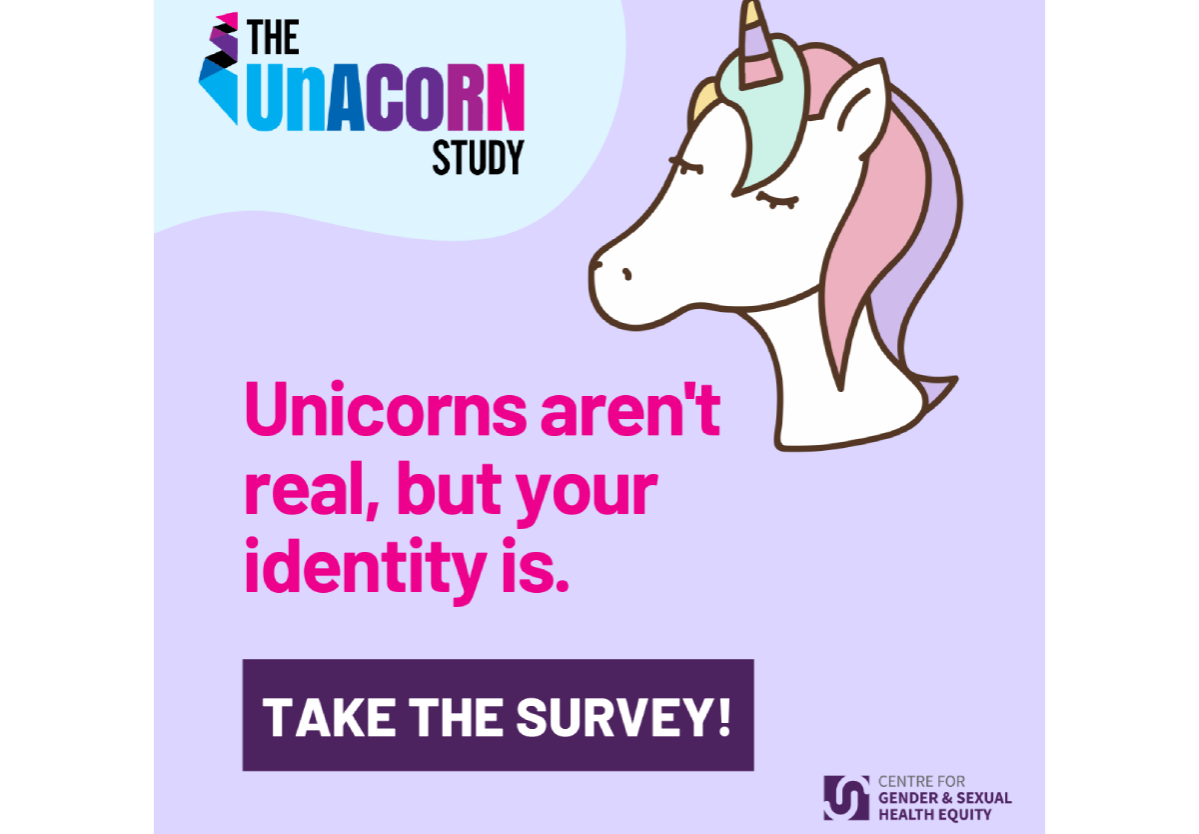
Image from the social media campaign.

Following SGM youth–specific recruitment recommendations from previous studies [11,23], our team designed the study’s website, social media campaigns, and distribution materials to emphasize the research project’s larger goal of contributing evidence toward efforts to make SGM youth feel safe and included (Figure 1). The promotional materials were distributed in French and English. Two separate surveys—one for each language—were programmed and harmonized in Qualtrics (Qualtrics, LLC). Both the French and English surveys were pilot-tested by team members and by people outside the research team who fit the UnACoRN eligibility criteria. Pilot testers were recruited through team members’ personal networks. They were asked to provide open feedback and were given a list of questions to answer about readability, programming errors, grammar, and content. Each pilot tester outside of the research team was provided a CAD $50 (the currency exchange rate for this study was US $1=CAD $1.25) honorarium for their work. The English version was pilot-tested by 8 non–team members and by 4 team members (SB, MG, SW, and TS). The French version was tested by one francophone outside of the research team, and by one bilingual team member (SB).

We applied branching logic to assign questions based on previous responses. Qualtrics bot detection was enabled to prevent automated and semiautomated responses. Following the recommendations from the developer, participants with a reCAPTCHA score below 0.5 were removed from the sample, as done in previous surveys [24].

### Sampling

The United States and Canada have wide differences in structural forms of stigma and legal protections for SGM people [25]. Therefore, including participants from the United States allowed us to explore a wider breadth of settings and circumstances that lead to affirmed or rejected identities and to prevent type II errors in future analyses of subsamples recruited through channels with a bigger proportion of people living in the United States. The target sample size for the web-based survey was 4000 people, chosen based on primary analyses of factors correlated with experiences of conversion therapy—assumed to occur at a prevalence of approximately 5%, based on previous research [10,26,27]—and a goal of ensuring at least 10 events per explanatory variable were included in a multivariable model with up to 20 covariates/levels (20 × 10 = 200 events; 200 / 0.05 = 4000). Inferential goals require adequate subsample sizes, while prevalence inquiries benefit from representativeness [28,29]. In this context, we aimed to recruit a Canadian subsample proportional to the distribution of the population of all provinces and territories.

### Ethical Considerations

This study was approved by the Simon Fraser University Research Ethics Board (#30000544). All participants who took part in the study consented to primary and secondary analyses of the research data. They were also offered the chance to enter a draw to win 1 of 15 CAD $100 gift cards or a stuffed toy unicorn. The information was collected using Qualtrics, stored inside a local secure server in Canada, and remained encrypted at all times. Participants were asked to provide contact information if they consented to be recontacted for future research.

### Recruitment

#### Venues

No gold standard method has been established to recruit SGM subjects [30]. In the absence of a sampling frame, combining as many theoretically distinct venues as possible is recommended [31]. Past studies have identified online recruitment as an effective tool to reach young SGM adults, with more explosive participation rates than in-person recruitment at community-specific venues [32]. Recent analyses suggest that platforms like Facebook or Instagram have become a safe space for youth trying to explore their sexual and gender identities [33]. Therefore, we combined conventional recruitment methods (eg, in-person) with online advertising, relying mostly on the latter. Survey responses and online campaign materials were monitored over time, following existing guidelines [34], to safeguard participants and involved staff (Multimedia Appendix 1) and to ensure adequate participation across geographic, age, gender modality (cis vs trans), gender identity (woman, nonbinary, and man), and sexual orientation strata. Recruitment occurred from March 16 to August 15, 2022, at the venues described below.

#### Allied Organizations

One of the authors (MG) recruited a small group (n=5) of high school, undergraduate, and graduate students who were connected with over 100 groups, organizations, and well-known allies of the 2S/LGBTQ community in Canada and some from the United States. They were provided with scripts and social media shareables. Key contacts within each group and organization were identified and asked—mainly through email and messages on social media—to share a post about the UnACoRN study or to recruit participants directly. Weekly briefings from the study team informed ongoing recruitment goals (eg, increasing diversity with regard to geography, race, cultural identity, and gender). We used this information to adjust recruitment efforts to target underrepresented sociodemographic subgroups. The response rate from the targeted organizations was low overall (approximately 15/127, 11.8%), although awareness of the study across community organizations likely bolstered the overall brand of the survey. It is also possible that some organizations shared the post through their communication channels without reporting back. As an example, we identified 7 organizations and groups outside of our initial list tweeting about the study. We could not calculate a response rate for this method because our tracking variables (eg, “heard about the study on Facebook”) could be attributed to multiple recruitment strategies.

#### Reddit and TikTok

Some subgroups or communities (eg, people who have detransitioned, that is, discontinued or reversed gender-affirming health care [16]) required specific recruitment methods to reach an adequate sample size. Therefore, we mapped approximately two dozen subreddits—community-specific forums on the Reddit social media website—and contacted their moderators to ask for permission to post the survey. Once we received confirmation, we posted a message tailored to the audience of the subreddit. Some subreddits had more relaxed rules and allowed us to post without explicit permission. Seven subreddits rejected or removed our posts, 7 never replied, and 9 allowed the authors to share the information. Based on available data regarding user views, we estimate we reached over 10,000 Reddit users from April to June; some users may have been counted twice in this estimate (ie, because they subscribed to several subreddits). Based on statistics provided by Reddit, we expected approximately half of this subsample to identify as gay men and a third to identify as bisexual. A TikTok user with over 25,000 followers, notably including members of the “detrans” community (ie, those who have detransitioned), also promoted the UnACoRN survey, reaching 1100 views.

#### Meta (Instagram and Facebook)

Advertisements on Meta were placed from April 13 to July 13. A total of 111 advertisements reached 128,896 people on Facebook and 414,976 on Instagram, resulting in 3291 (2.55%) and 14,888 (3.59%) link clicks, respectively. Meta identified 4707 as “male,” 10,576 as “female,” and 2899 as “uncategorized”; these data are independent of that provided by the participants in the survey and cannot be linked or verified. Advertisements were limited by age (15 to 29 years); most (9250/15,283, 60.52%) of the visitors were younger than 18 years. These visitors had a lower cost per click ($0.21 compared to $0.29 for all other groups), and Meta allocated a greater proportion of the budget to this group to optimize the campaign results, even though this group had a lower click-through rate (0.87%), than the 18-to-24-year (2.87%) or 25-years-and-older (3.59%) age groups (absolute values were not available from Meta). To compensate for these effects, we ran some advertisements specifically for people aged 18 years or older.

#### Pornhub

A total of 5 advertisements were run on Pornhub, targeting heterosexual (61.7% of the budget) and gay men in Canada from May 5 to May 30. We used customized work-safe graphics and sexual orientation–specific keywords. The total number of impressions was close to 5 million (39.3% were gay). The ad broker did not provide data on actual reach (ie, the specific number of visitors).

#### Bus Advertisements

We started running a set of 25 interior transit card advertisements (ie, advertisements inside buses) in Ottawa and Halifax on May 16. These 2 cities were selected because they included the majority (56%) of the population aged 15 to 29 years among the 10 cities in 5 provinces where the advertising company operated [35]. Based on data shared by the company, 33,400 youth may have seen the advertisements during the 12-week bus campaign.

#### Media Interviews

Two team members (SB and MG) made appearances on a radio show and a podcast. SB promoted the survey on a French-language morning radio show in British Columbia. MG spoke on a niche queer-focused Canadian podcast. Neither of these activities resulted in any immediately noticeable uptick in participation.

### Analysis

We used counts and proportions to describe our sample recruitment process, including a comparison between participants who started the survey (ie, provided sociodemographic data) and those who finished the survey. For participants who lived in Canada, we also estimated the proportional difference relative to the population estimates provided by Statistics Canada [36]. We also analyzed the metadata provided by Bitly, Meta, TrafficJunky (the Pornhub ad broker), and Qualtrics and combined it with participants’ input regarding recruitment. We displayed the results using a Sankey graph. Our paid campaign was assessed through the completion rate and cost of completed survey metrics. Finally, we reported the proportion of SGM participants by declared recruitment venue and described the unique challenges we identified early in the campaign.

## Results

### Participant Characteristics

A total of 16,533 people accessed the English (14,551, 88%) and French (1982, 12%) surveys. We excluded 205 individuals who were either categorized as “spam” or “bots” by the reCAPTCHA score Qualtrics function (the average reCAPTCHA score in the full sample was 0.94). An additional 128 visitors did not provide consent, yielding 16,200 valid responses. A further 6521 of the 16,200 people who consented abandoned the survey upon encountering some questions that would allow the research team to validate their identity in case they accidentally closed the browser. As a result, a total of 9679 people provided research data and were thus available as participants in further analytic samples. This sample included 7037 residents of Canada, distributed across all provinces and territories (Table 1), and 2521 residents from all US states and the District of Columbia. The overall completion rate was 59.7% (5777/9679) (Multimedia Appendix 2); of those who completed the survey, 1753 of 5777 (30.34%) agreed to be followed up. The median time for survey completion was 33.1 minutes.

**Table 1:** Comparison of UnACoRN^a^ participants (n=7059) from Canada and Statistics Canada’s estimates of the proportion of the total population (n=7,150,260) for people aged 15 to 29 years by province or territory, age, and urban/rural area. The proportion of missing answers includes participants who decided to skip the question.

Sociodemographic characteristics		Canada, n (%)	UnACoRN, n (%)	Difference in proportion, percentage points
**Province or territory (n=6122)^b^**				
	Newfoundland and Labrador	83,148 (1.16)	126 (2.06)	0.90
	Prince Edward Island	33,818 (0.47)	21 (0.34)	–0.13
	Nova Scotia	177,402 (2.48)	191 (3.12)	0.64
	New Brunswick	129,250 (1.81)	172 (2.81)	1
	Quebec	1,466,271 (20.51)	1444 (23.59)	3.08
	Ontario	2,935,514 (41.05)	2100 (34.3)	–6.75
	Manitoba	277,583 (3.88)	309 (5.05)	1.17
	Saskatchewan	221,239 (3.09)	276 (4.51)	1.42
	Alberta	835,667 (11.69)	623 (10.18)	–1.51
	British Columbia	963,715 (13.48)	837 (13.67)	0.19
	Yukon	7542 (0.11)	10 (0.16)	0.05
	Northwest Territories/Nunavut	19,111 (0.26)	13 (0.21)	–0.05
**Age group (n=6498)^c^ (years)**				
	15 to 19	2,057,449 (28.77)	4135 (63.63)	34.86
	20 to 24	2,452,701 (34.3)	1386 (21.33)	–12.97
	25 to 29	2,640,110 (36.92)	977 (15.04)	–21.88
**Area (n=6122)^b^**				
	Urban	6,189,855 (86.57)	5445 (88.94)	2.37
	Rural	960,405 (13.43)	677 (11.06)	–2.37

^a^UnACoRN: Understanding Affirming Communities, Relationships, and Networks.

^b^Missing answers for UnACoRN: 13.3% (937/7059).

^c^Missing answers for UnACoRN: 7.9% (561/7059).

Among 8912 Canada and US participants who provided demographic information, 5660 (68.5%) were aged 15 to 19 years, 1474 (17.9%) were aged 20 to 24 years, and 1123 (13.6%) were aged 25 to 29 years. In terms of gender, 3412 (38.3%) identified as women, 2827 (31.7%) as nonbinary, and 2183 (24.5%) as men; 5820 (65.3%) either were classified as trans (ie, using the 2-step method based on sex assigned at birth and current gender identity [37]) or self-reported trans experience (ie, the 1-step method [37]); 3026 (34%) of participants identified as bisexual, 2541 (28.5%) as queer, 1762 (19.8%) as pansexual, 1672 (18.8%) as asexual, 1398 (15.7%) as lesbian, 1158 (13%) as gay, 878 (9.9%) as heterosexual, and 757 (8.5%) as fluid; 561 (6.3%) provided other responses for sexual identity. Overall, 8472/8912 participants or 95.1% of the sample were from SGM populations (Multimedia Appendix 2).

### Campaign Analysis

**Figure 2:**
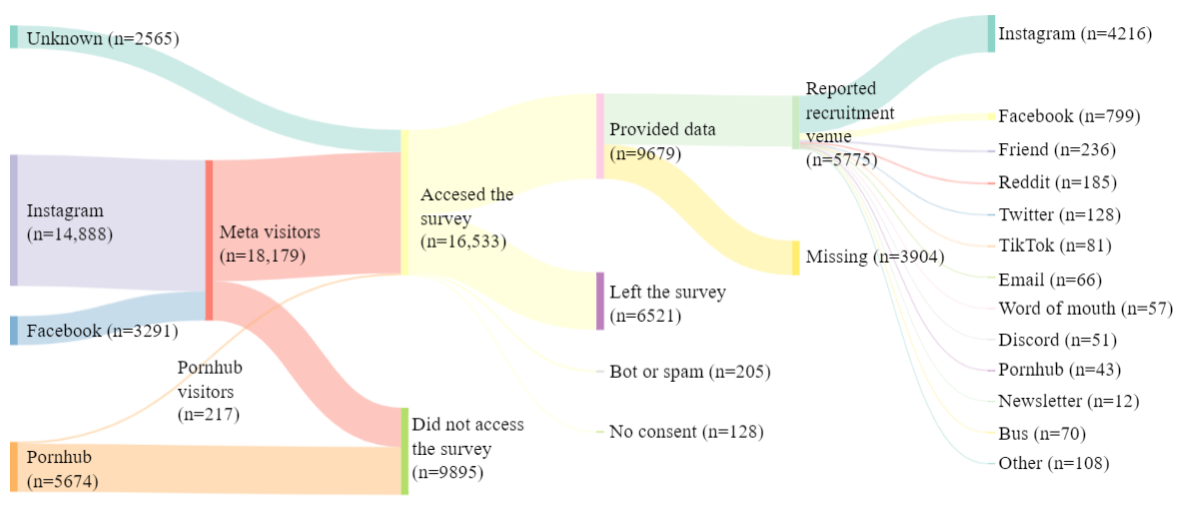
Recruitment sources (left-most block) and declared sources of participation (right-most block) in the Understanding Affirming Communities, Relationships, and Networks (UnACoRN) survey.

Separate Qualtrics collection links were used for different recruitment venues. We also asked participants how they heard about the survey. Both analyses showed the majority of participants were likely recruited through Meta (13,741/16,533, 83.1%)—the parent company of Instagram and Facebook—mostly through Instagram (Figure 2). The remainder of the visitors came from unidentified sources (2565/16,533, 15.5%) and Pornhub (217/16,533, 1.31%).

Based on the Qualtrics collection links and the information provided by the 9679 survey participants who provided research data, we estimate that 8610 (88.96%) reached the survey through one of our paid advertisements (Table 2). Of the remainder (1069), 185 (3.2%) participants said they reached the survey through Reddit and 81 (1.4%) through TikTok (Figure 2). Overall, the cost per survey was lower for Meta than Pornhub or the bus advertisements (Table 2). Similarly, the proportion of visitors who started the survey was higher for Meta (8492/18,179, 46.7%) than Pornhub (58/5674, 1.02%). The overall average cost per completed survey, including those who reached the survey through unpaid venues, was CAD $1.48.

**Table 2:** Description of costs and results of the paid campaign and participants who started or completed the Understanding Affirming Communities, Relationships, and Networks (UnACoRN) survey. Overall, 8607 participants started the survey and 5777 completed it, including completed surveys from unpaid venues. The total cost of the campaign was CAD $8571.58^a^ and the overall cost per completed survey was CAD $1.48.

Venue	Estimated audience, n	Total visitors, n	Started the survey^b^, n (%)	Completed the survey^b^, n (%)	Cost, CAD $	Cost per completed survey, CAD $
Facebook	128,896	3291	1269 (38.56)	805 (24.46)	882.23	1.02
Instagram	414,976	14,888	7223 (48.52)	4297 (28.86)	3414.42	0.79
PornHub	5,129,417^c^	5674	58 (1.02)	29 (0.51)	836.93	28.86
Bus advertisements	N/A^d^	N/A	57^e^	57^e^	3438	60.31

^a^CAD $1.25=US $1.

^b^Proportion of visitors.

^c^Pornhub provides data on impressions, not reach.

^d^N/A: not applicable.

^e^Total number of visitors and percentage were not available.

### Recruitment Venues for Hard-To-Reach Populations

The proportion of sexual minorities was >90% among participants from all online venues; the proportion was at the same level among those reporting the bus advertisements as their recruitment venue (64/70, 91%). However, the proportion of gender minorities varied from 47% (20/43, Pornhub) to 93% (75/81, TikTok) according to the recruitment venue (Table 3). Few participants identified as cis-het during the early stages of the campaign. We also saw an overrepresentation of people younger than 20 years and of participants who self-identified as White compared to what we initially expected. While adjustments to the campaign materials (Multimedia Appendix 3) seemed to reduce these gaps—the proportion of English-speaking participants identifying as men increased steadily from 14.2% (404/2845) in April to 20.5% (405/1976) in July—SGM status, age, and cultural identity imbalances remained in our final sample.

**Table 3:** Gender minority status and sexual minority status by recruitment venue.

	Facebook (n=799), n, (%)	Instagram (n=4216), n, (%)	Reddit (n=185), n, (%)	TikTok (n=81), n, (%)	Pornhub (n=43), n, (%)	Bus (n=70), n, (%)
Gender minority	516 (64.58)	3033 (71.94)	143 (77.30)	75 (92.59)	20 (46.51)	42 (60)
Sexual minority	736 (92.12)	4033 (95.66)	167 (90.27)	75 (92.59)	42 (97.67)	64 (91.43)

## Discussion

### Implications

This study documents campaign strategies, designs, procedures, and outcome measures for recruiting SGM populations in Canada and the United States. Our mixed (online and in-person) approach led to the successful recruitment of SGM participants, particularly in Canada, where our sample resembled the distribution of the population in all Canadian provinces and territories. The UnACoRN results are comparable to those of the Writing Themselves in 4 survey in Australia [7] in terms of geographic distribution (ie, urban vs rural), sexuality diversity, and gender diversity, although the latter does not include cis-het participants, a feature that differentiates our survey from others targeting SGM youth [8,9]. Likewise, the inclusion of online niche venues—including pornography websites—has remained mostly unexplored in previous research at the national level.

Our campaign analysis offers insights into 3 online venues (Instagram, Facebook, and Pornhub) and one in-person venue (bus advertisements), finding that social media use was more advantageous in terms of cost-effectiveness and overall recruitment goals. We recruited the most participants at a very low cost on Facebook (CAD $1.02 per survey) and Instagram (CAD $0.79 per survey). These results could be attributed to many factors, including greater internet penetration and adoption in recent years, a growing share of users of Meta platforms compared to other social media over time, a tailored campaign that used 2S/LGBTQ-friendly design and rhetoric, and adaptive recruitment strategies.

Our overall cost of CAD $1.48 per survey is low compared to most studies recruiting through Facebook, which have had an average cost per completed participation of CAD $6.79, with a range from CAD $1.36 to $110 [38]. Past SGM youth surveys at the national level have not reported this metric based on advertising venue costs. Many surveys of SGM populations have, however, offered individual incentives or honoraria to survey participants, which can serve as one point of comparison for cost-effectiveness purposes. For example, the Generations project in the United States provided a US $25 gift card to each respondent [9]. Online recruiting with tailored advertisements remains a cost-effective alternative to samples derived from general population frames (eg, school-based research), particularly in countries where this is counterproductive due to religious or political opposition to SGM individuals [39].

### Strengths and Limitations

In 2021, Canada was the first country to ask about gender identity in a census. Approximately 55,000 Canadians aged 15 to 29 years were identified as gender minorities by a member of their household in the first census in the world that gathered data on gender identity [40]. If we assume these census data are an accurate measure of gender identity, UnACoRN would have gathered data from approximately 5.6% of this subpopulation, with a distribution that resembled that of the Canadian provinces and territories. Notably, many gender minorities may be reluctant to disclose their identities in the context of pervasive antitrans stigma [41], leading some researchers to suggest that census counts are likely substantially underestimating the true population size [42]. Nonetheless, in the absence of reliable SGM population data at the national level, UnACoRN offers a unique opportunity to understand SGM youth in Canada and the United States, particularly those younger than 19 years. Moreover, we took an intersectional approach that will allow us to study populations that have been understudied, like young bisexual women and transgender individuals [43].

While SGM populations have historically been considered “hidden,” online recruitment is making it easier to obtain data for research purposes, particularly among youth. Online recruitment is also hypothesized to reduce selection bias for less-connected SGM people (ie, those in rural contexts or dealing with mental health issues) [43,44]. Our sizable sample, extensive geographic coverage in Canada and the United States, and high cost-effectiveness compared to previous studies confirm this. However, not all online venues had a low cost per completed survey: a much lower proportion of visitors from Pornhub started the survey compared to Meta. Significant differences in recruitment by sexual or gender minority status are likely a by-product of recruitment strategies targeting identity-related communities, be it through posting on specific channels (eg, a detrans subreddit), budget allocation (automatically allocated by advertising brokers), or messaging framing.

While advantageous, online methods had the drawback of excluding those who were less likely to use the internet or, in the case of our study, social media. Therefore, our study is limited in that it is not possible for us to anticipate in which ways our populations differ from SGM youth overall. To address this, we also conducted in-person recruitment. Future studies should investigate to what extent UnACoRN estimates compare to population-based samples like the Canadian and Community Health Survey. Another limitation of our study is its cross-sectional nature, which limits inferential goals to those instruments that account for the temporality of the construct. However, even in those cases, recall bias might be an issue. Moreover, sexual and gender identity changes over time in both adults and adolescents [43]. We plan to conduct follow-up studies (eg, UnACoRN 2.0) among those who consented to recontact to monitor changes in our sample.

### Conclusion

In conclusion, online recruitment for SGM youth remains an active area of methodological experimentation and improvement. Our work has contributed by providing estimates showing that social media, specifically Instagram, are the most cost-effective venue in our target population when compared to bus advertisements and other websites. We also cast a wider net by using promotion on content-specific channels, which further contributed to obtaining a sexual- and gender-diverse sample. UnACoRN joins other national-level online surveys that monitor the health and well-being of SGM youth in North America. The geographic distribution of participants residing in Canada resembled the geographic distribution of the national subpopulation of a similar age and allowed for adequate estimation of inferential goals that will contribute knowledge to support the 2S/LGBTQ community.

Researchers using online recruitment strategies should be aware of the differences in campaign management each website or social media offers and be prepared to engage with their framing (content selection and delivery) to correct any imbalances derived from these differences. Those who focus on SGM populations should consider that 2S/LGBTQ-friendly campaigns might deter participation from cis-het people, if they are relevant to the research design. Finally, researchers with limited resources might select fewer venues with a lower cost per completed survey or venues that appeal more to their specific audience, if needed.

## Appendix

Multimedia Appendix 1 Supplementary Table 1. Recruitment and monitoring criteria results.

Multimedia Appendix 2 Supplementary Table 2. Comparison of participants who started the survey and filled the sociodemographic section and those who finished the survey.

Multimedia Appendix 3 UnACoRN Survey: Campaign design changes over time.

## Abbreviations

2S/LGBTQ: two-spirit, lesbian, gay, bisexual, transgender, and queer
Cis-het: cisgender-heterosexual
SGM: sexual and gender minority
SOGIE: sexual orientation, gender identity, and expression
SOGIECE: sexual orientation and gender identity and expression change efforts
UnACoRN: Understanding Affirming Communities, Relationships, and Networks

## Data Availability

Due to the nature of this research, participants in this study did not agree for their data to be shared publicly. The data that support the findings of this study are available from the principal investigator (TS) upon reasonable request. More information about the survey can be found on the survey website (https://unacorn.ca/).
