# Supplementary material for: Sampling Sexual and Gender Minority Youth With UnACoRN (Understanding Affirming Communities, Relationships, and Networks): Lessons From a Web-Based Survey
Source: J Med Internet Res. 2023 Jan 12;25:e44175. doi: 10.2196/44175 (PMC9893884; doi:10.2196/44175)
Supplement: Multimedia Appendix 1 [file jmir_v25i1e44175_app1.docx]

## Multimedia Appendix 1

Supplementary Table 1. Recruitment and monitoring criteria results.

| **Criteria** | **Answer** | **Notes** |
| --- | --- | --- |
|  |  |  |
| **Monitoring** |  |  |
| ***Page administration*** |  |  |
| At least two research team members were assigned as administrators of the public page hosting the advertisement. | Yes |  |
| The research team used a shared decision-making process to monitor and respond to comments made on posts and ads. | Yes | The responses were tailored mostly towards negative comments. |
| ***Notifications*** |  |  |
| All administrators downloaded the Facebook app to their mobile phones prior recruitment | Yes | We downloaded the Meta Business app. |
| Notifications were monitored based on a predetermined schedule (eg, hourly or daily) | Yes | We monitored on an ongoing basis. At least daily. |
| ***Recruitment Cycle Duration*** |  |  |
| Advertisements were posted for a maximum of 7 days per cycle. | No | Advertisements were monitored and removed or turned off based on comparative cost per click within an ad set. However, they were deactivated if the stuff was on vacation. |
| ***Inclusion and Exclusion Terms*** |  |  |
| Researchers used inclusion terms based on study criteria. | No | Advertisements were tailored through design (e.g. colors, phrasing), not keywords. |
| Researchers used exclusion terms based on study criteria to guard against the inclusion of social media users who may engage in digital harassment and cyberbullying directed at the intended study population. | No |  |
| ***Public page settings and moderation*** |  |  |
| Researchers restricted who can post directly to a public page | Yes |  |
| Researchers set the profanity filter to high | Yes |  |
| **Responding** |  |  |
| The research team reviewed the privacy, page moderation, profanity, and reporting features prior to recruitment | Yes |  |
| Researchers hid/blocked comments if the content constituted digital harassment or cyberbullying. | Yes | Comments were deleted if the content was offensive or profane. |
| For repeated offenses, users were blocked and reported to Facebook. | NA | We did not have repeated offenders |
| **Reporting** |  |  |
| All negative comments and interactions were screenshotted and reported to the research PI by email within 24 hours. | Yes |  |
| The PI documented negative interactions and comments in a secure, password-protected electronic location. | Yes |  |
| The research team reported potentially dangerous interactions or comments (eg, emotional or psychological harm, threatened physical harm) to their IRB within 48h or maximum a week. | NA | We did not receive this type of comments. |
| ***Research and study stuff*** |  |  |
| Negative interactions and comments were debriefed at least weekly to mitigate secondary traumatic stress | Yes | We discussed all negative comments, but there were very few of them. |
| The research team was provided with contact information for affordable and/or free and sliding scale psychological services | No |  |
| ***General public*** |  |  |
| The “About: section of the social media page contains a list of available localized support resources (with contact information) for individuals to access for support and coping with cyberbullying. | No | The research website contains a list of available localized support resources for individuals to access for support and coping with cyberbullying. |
