# Supplementary material for: Sampling Sexual and Gender Minority Youth With UnACoRN (Understanding Affirming Communities, Relationships, and Networks): Lessons From a Web-Based Survey
Source: J Med Internet Res. 2023 Jan 12;25:e44175. doi: 10.2196/44175 (PMC9893884; doi:10.2196/44175)
Supplement: Multimedia Appendix 2 [file jmir_v25i1e44175_app2.docx]

## Multimedia Appendix 2

Supplementary Table 2. Comparison of participants who started the survey and filled the sociodemographic section and those who finished the survey.^a^

|  | **Started the survey (n=8,912)** | | **Completed the survey (n=5,777)** | |
| --- | --- | --- | --- | --- |
|  | **n** | **(%)^b^** | **n** | **(%)^b^** |
|  |  |  |  |  |
| **Language** |  |  |  |  |
| English | 7880 | 88.4 | 5147 | 89.1 |
| French | 1032 | 11.6 | 630 | 10.9 |
| **Gender** |  |  |  |  |
| Woman | 3412 | 38.3 | 2220 | 38.4 |
| Non-binary | 2827 | 31.7 | 1843 | 31.9 |
| Man | 2183 | 24.5 | 1483 | 25.7 |
| Unsure | 1320 | 14.8 | 849 | 14.7 |
| Genderfluid | 1299 | 14.6 | 804 | 13.9 |
| Genderqueer | 1194 | 13.4 | 775 | 13.4 |
| Agender | 741 | 8.3 | 490 | 8.5 |
| Third Gender | 70 | 0.8 | 46 | 0.8 |
| Detrans | 50 | 0.6 | 25 | 0.4 |
| None/Other | 477 | 5.4 | 319 | 5.5 |
| **Gender Minority^c^** |  |  |  |  |
| Yes | 6184 | 69.4 | 4018 | 69.6 |
| No | 2728 | 30.6 | 1759 | 30.4 |
| **Sexuality** |  |  |  |  |
| Bisexual | 3026 | 34.0 | 1924 | 33.3 |
| Queer | 2541 | 28.5 | 1714 | 29.7 |
| Pansexual | 1762 | 19.8 | 1149 | 19.9 |
| Asexual | 1672 | 18.8 | 1134 | 19.6 |
| Lesbian | 1398 | 15.7 | 929 | 16.1 |
| Unsure | 1261 | 14.1 | 794 | 13.7 |
| Gay | 1158 | 13.0 | 784 | 13.6 |
| Heterosexual | 878 | 9.9 | 563 | 9.7 |
| Fluid | 757 | 8.5 | 463 | 8.0 |
| Indigiqueer | 36 | 0.4 | 22 | 0.4 |
| None/Other | 561 | 6.3 | 379 | 6.6 |
| **Sexual Minority^d^** |  |  |  |  |
| Yes | 8367 | 93.9 | 5404 | 93.5 |
| No | 545 | 6.1 | 373 | 6.5 |
| **Sexual or Gender Minority^e^** |  |  |  |  |
| Yes | 8472 | 95.1 | 5495 | 95.1 |
| No | 440 | 4.9 | 282 | 4.9 |
| **Age** |  |  |  |  |
| 15-19 | 5660 | 68.5 | 3540 | 65.6 |
| 20-24 | 1474 | 17.9 | 1030 | 19.1 |
| 25-29 | 1123 | 13.6 | 824 | 15.3 |
| **Country** |  |  |  |  |
| Canada | 6495 | 73.2 | 4147 | 72.0 |
| United States | 2376 | 26.8 | 1614 | 28.0 |
| **Area** |  |  |  |  |
| Urban | 5098 | 89.0 | 3310 | 89.2 |
| Rural | 632 | 11.0 | 400 | 10.8 |
| **Race** |  |  |  |  |
| White | 7340 | 82.4 | 4847 | 83.9 |
| Asian | 785 | 8.8 | 482 | 8.3 |
| Indigenous | 565 | 6.3 | 335 | 5.8 |
| Hispanic | 448 | 5.0 | 285 | 4.9 |
| Black | 355 | 4.0 | 209 | 3.6 |
| Pacific Islander | 38 | 0.4 | 19 | 0.3 |
| None/Other | 341 | 3.8 | 207 | 3.6 |
| Don’t know | 228 | 2.6 | 129 | 2.2 |
| Prefer not to answer | 131 | 1.5 | 68 | 1.2 |
| **Cultural identity** |  |  |  |  |
| European | 6225 | 69.8 | 4132 | 71.5 |
| Indigenous | 841 | 9.4 | 530 | 9.2 |
| Hispanic/Latinx | 597 | 6.7 | 371 | 6.4 |
| East Asian | 439 | 4.9 | 264 | 4.6 |
| African | 318 | 3.6 | 186 | 3.2 |
| Southeast Asian | 257 | 2.9 | 161 | 2.8 |
| South Asian | 226 | 2.5 | 137 | 2.4 |
| Middle Eastern | 220 | 2.5 | 133 | 2.3 |
| Pacific Island | 40 | 0.4 | 25 | 0.4 |
| None/Other | 797 | 8.9 | 527 | 9.1 |
| Prefer not to answer | 682 | 7.7 | 410 | 7.1 |

^a^Participants were also allowed to select multiple options for sexuality, gender identity, race, and ethnicity.

^b^For questions that were optional, the proportion of missing answers were 7.35% for age, 0.46% for country, and 35.70% for area among those who started the survey and 6.63% for age, 0.28% for country, and 35.78% for area among those who completed the survey.

^c^Participants were categorized as a gender minority if they (1) identified as a person of trans experience or were unsure about it, (2) their gender identity was different from the sex/gender they were assigned at birth, (3) they were born with a variation in their physical sexual characteristics, or (4) described their gender identity as something other than binary (i.e., man or woman).

^d^Participants were categorized as a sexual minority if they expressed any type of non-heterosexual sexual attraction or contact and/or described their sexuality as non-heterosexual.

^e^Participants were categorized as a sexual or gender minority if they were either a gender minority or sexual minority.
