# Supplementary material for: Sampling Sexual and Gender Minority Youth With UnACoRN (Understanding Affirming Communities, Relationships, and Networks): Lessons From a Web-Based Survey
Source: J Med Internet Res. 2023 Jan 12;25:e44175. doi: 10.2196/44175 (PMC9893884; doi:10.2196/44175)
Supplement: Multimedia Appendix 3 [file jmir_v25i1e44175_app3.pdf]

UnACoRN Survey:
Campaign design changes over time

Jorge Andrés Delgado-Ron
Thiyaana Jeyabalan
Sarah Watt
Stéphanie Black
Martha Gumprich
Travis Salway.

REAFFIRM COLLABORATIVE

Coming soon...

- We kickstarted with a teaser campaign that positioned the UNACORN logo.

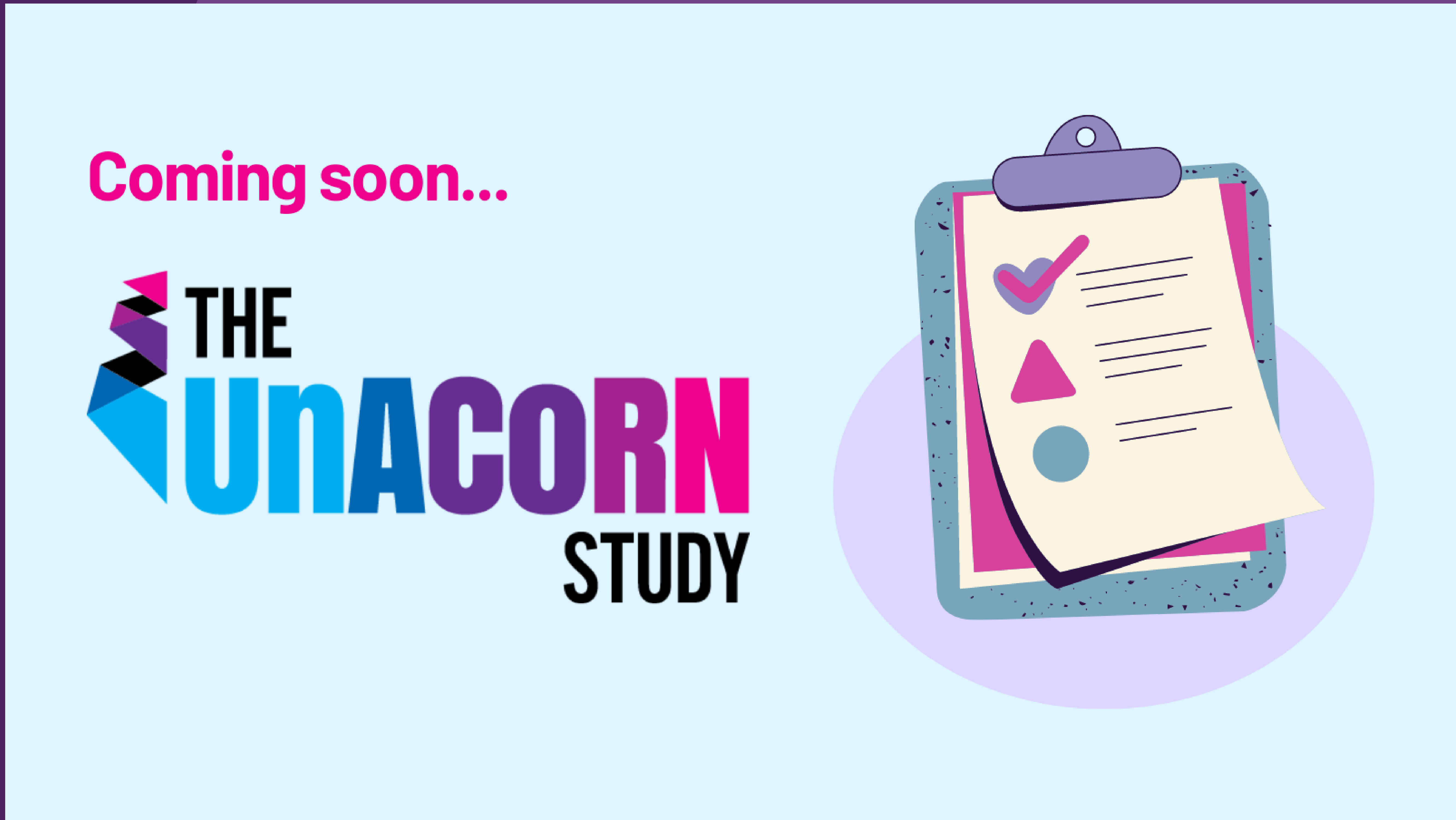

REAFFIRM COLLABORATIVE

Framing

- Our colours and messages aimed to be inclusive and make the 2S/LGBTQ community feel welcomed and safe.

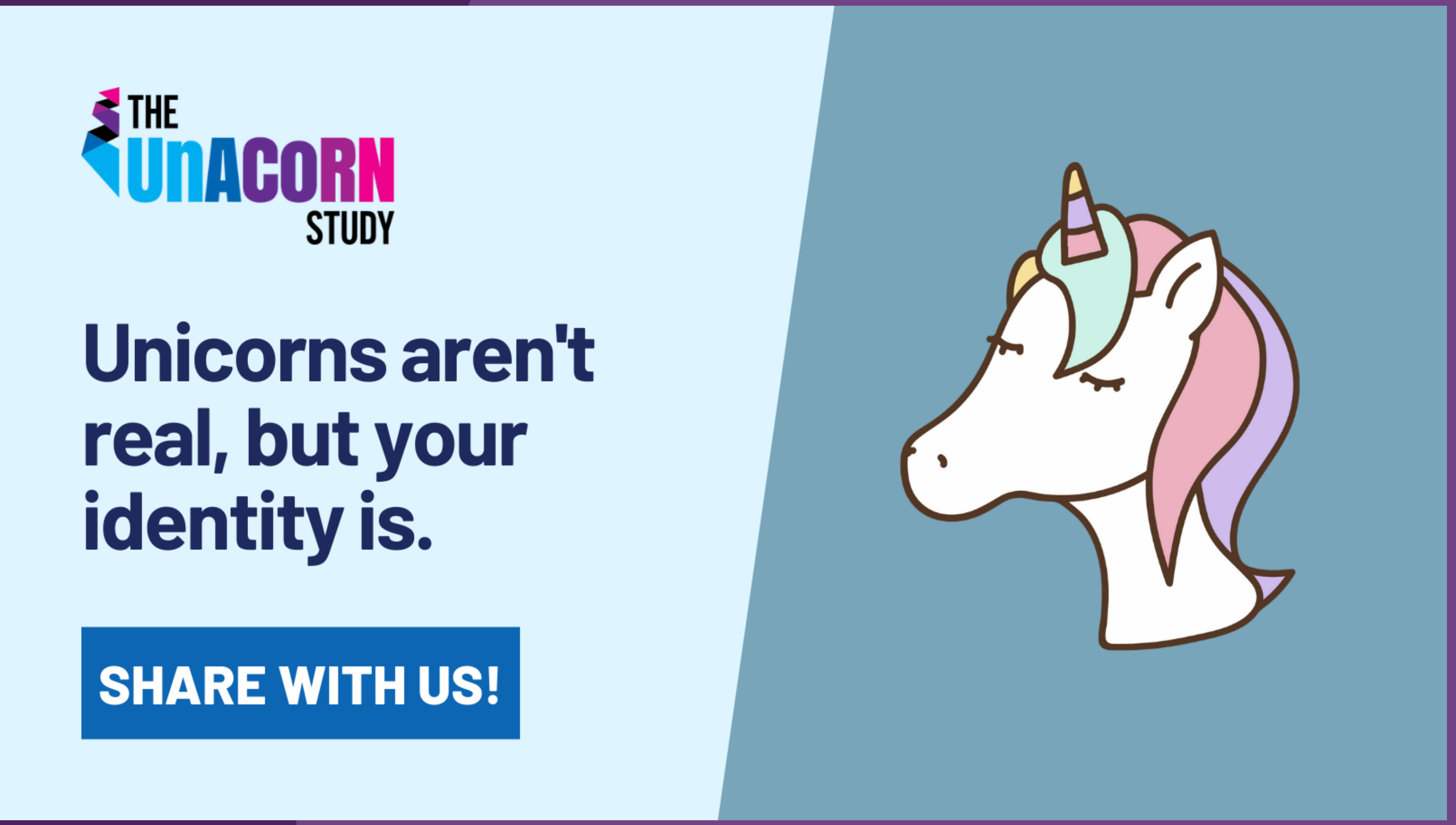

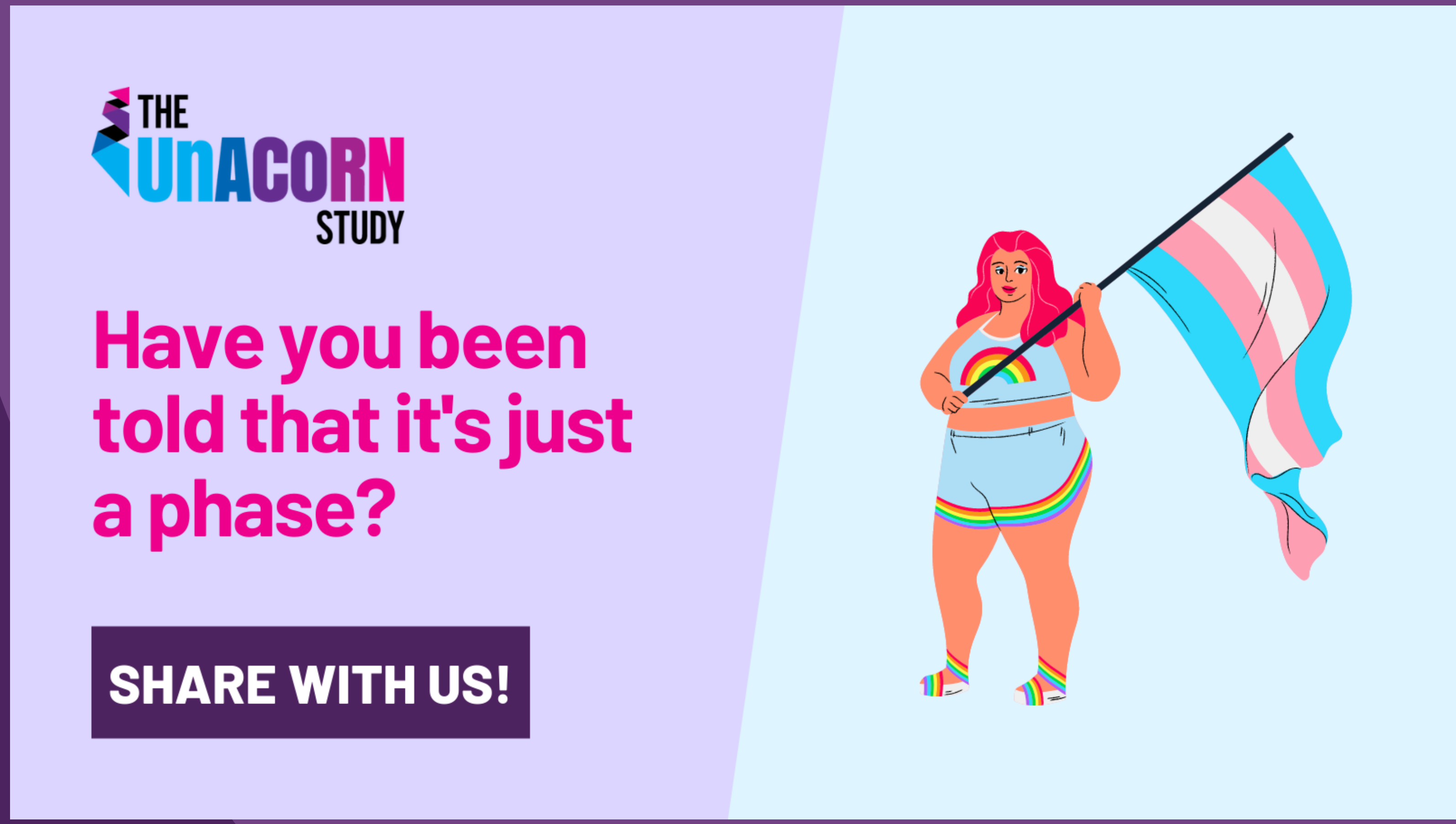

REAFFIRM COLLABORATIVE

Section-specific content

- We also used thematic content to appeal participants whose background matched key exposures explored in the survey

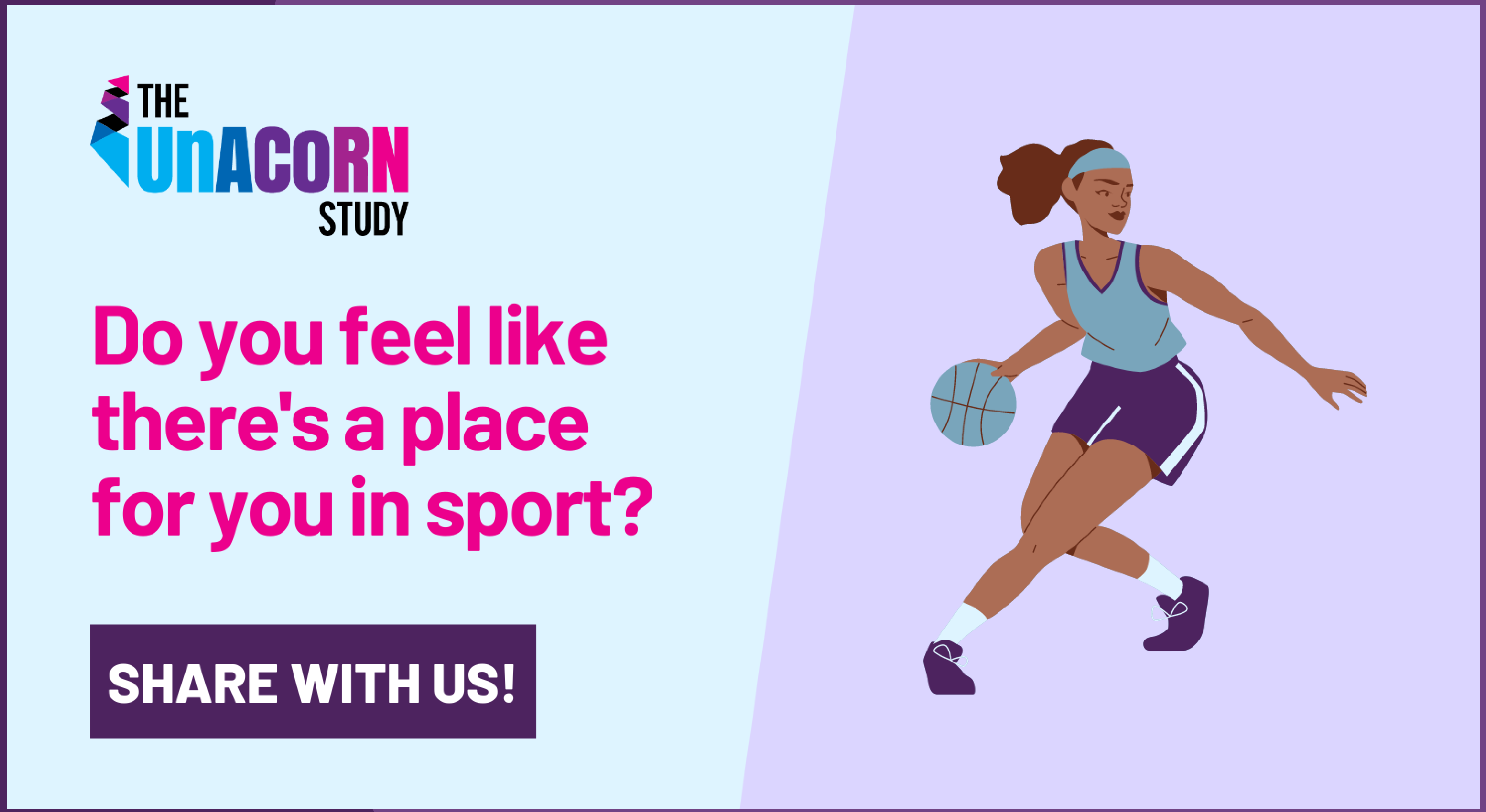

REAFFIRM COLLABORATIVE

Section-specific content

- We also used thematic content to appeal participants whose background matched key exposures explored in the survey

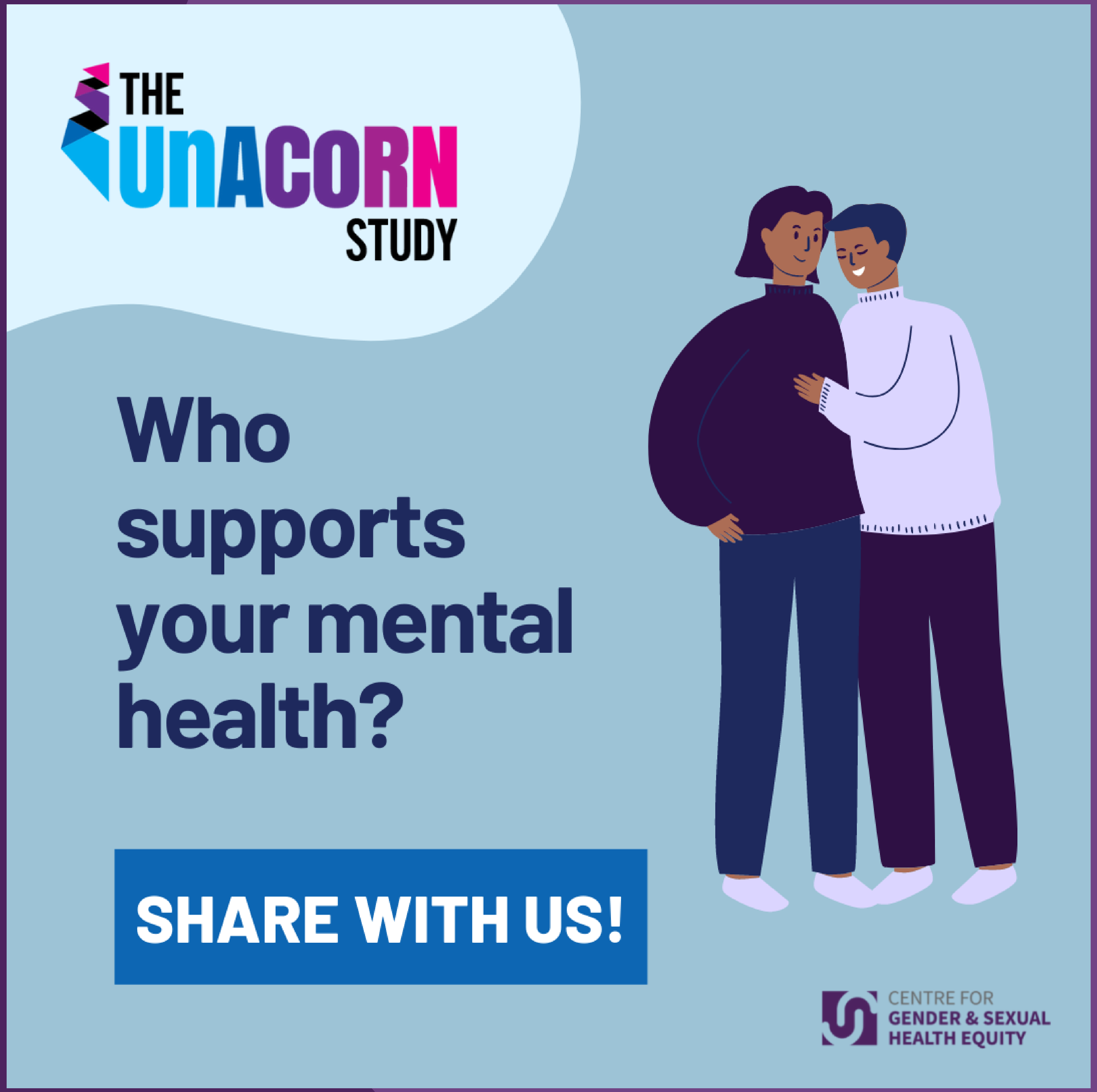

REAFFIRM COLLABORATIVE

Closing gaps

- However, it became evident that we needed to address some gaps, mainly for men

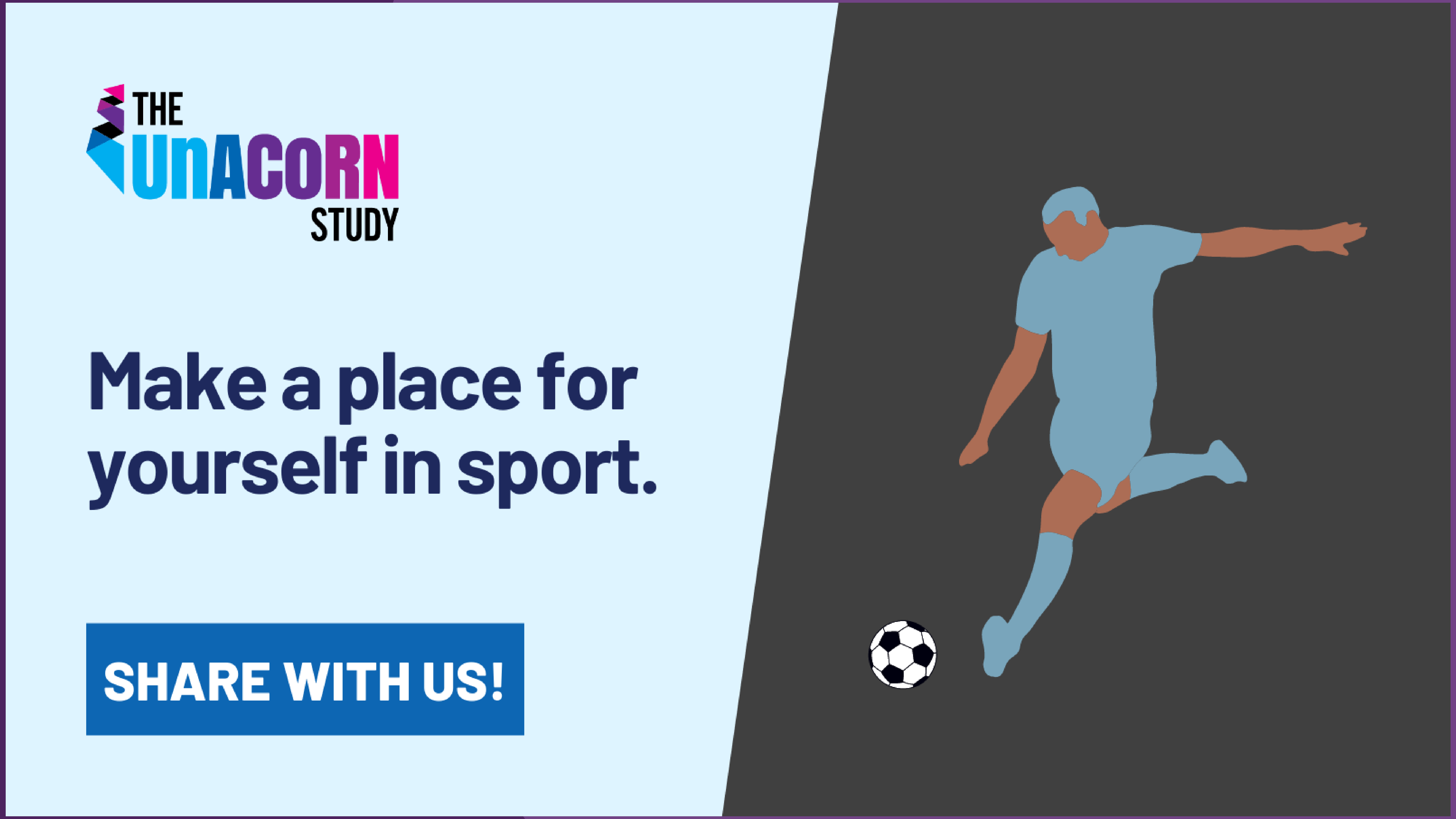

REAFFIRM COLLABORATIVE

Closing gaps

- However, it became evident that we needed to address some gaps, mainly for men, cis-het participants

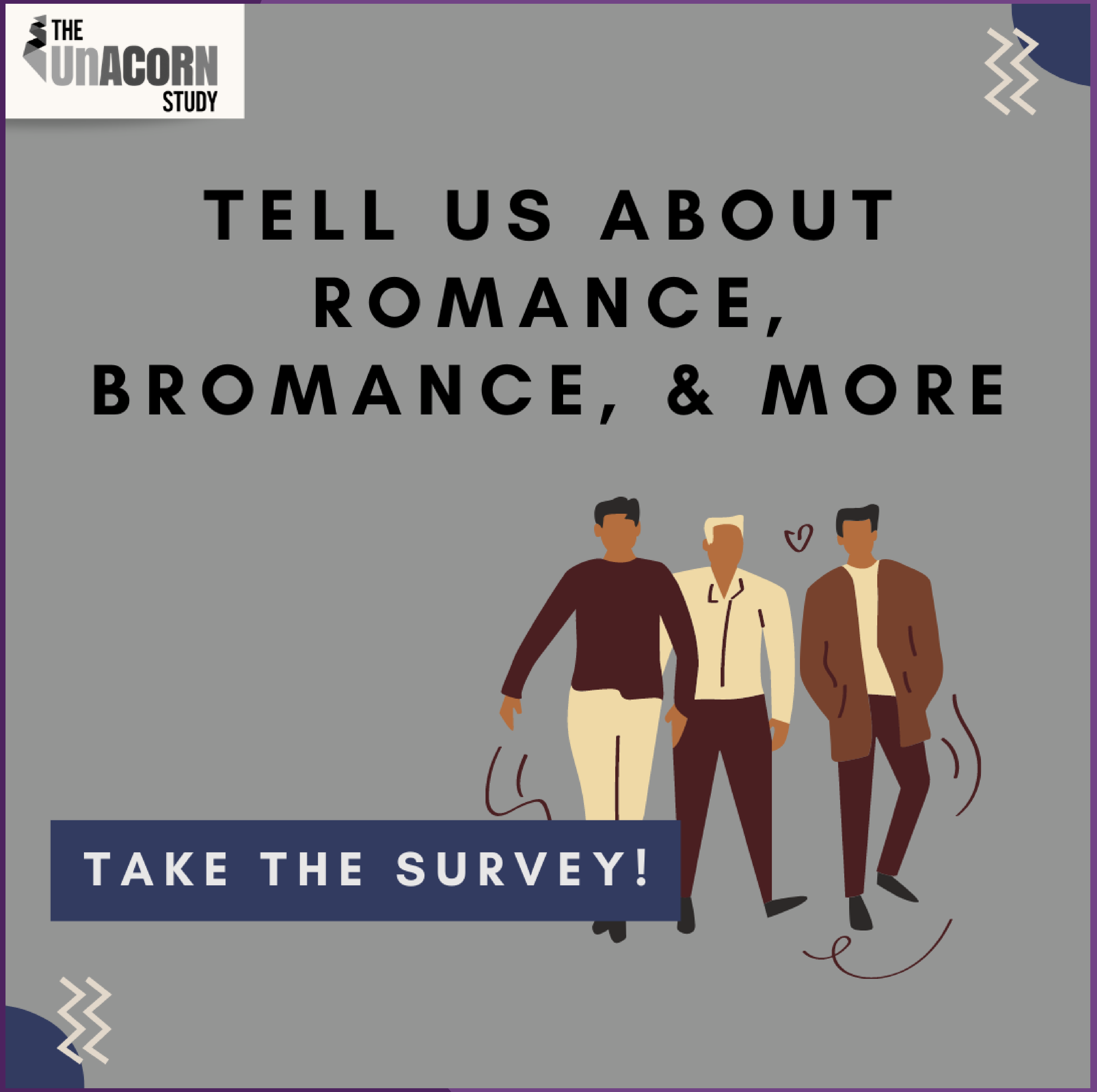

REAFFIRM COLLABORATIVE

Closing gaps

- However, it became evident that we needed to address some gaps, mainly for men, cis-het participants, and older age groups

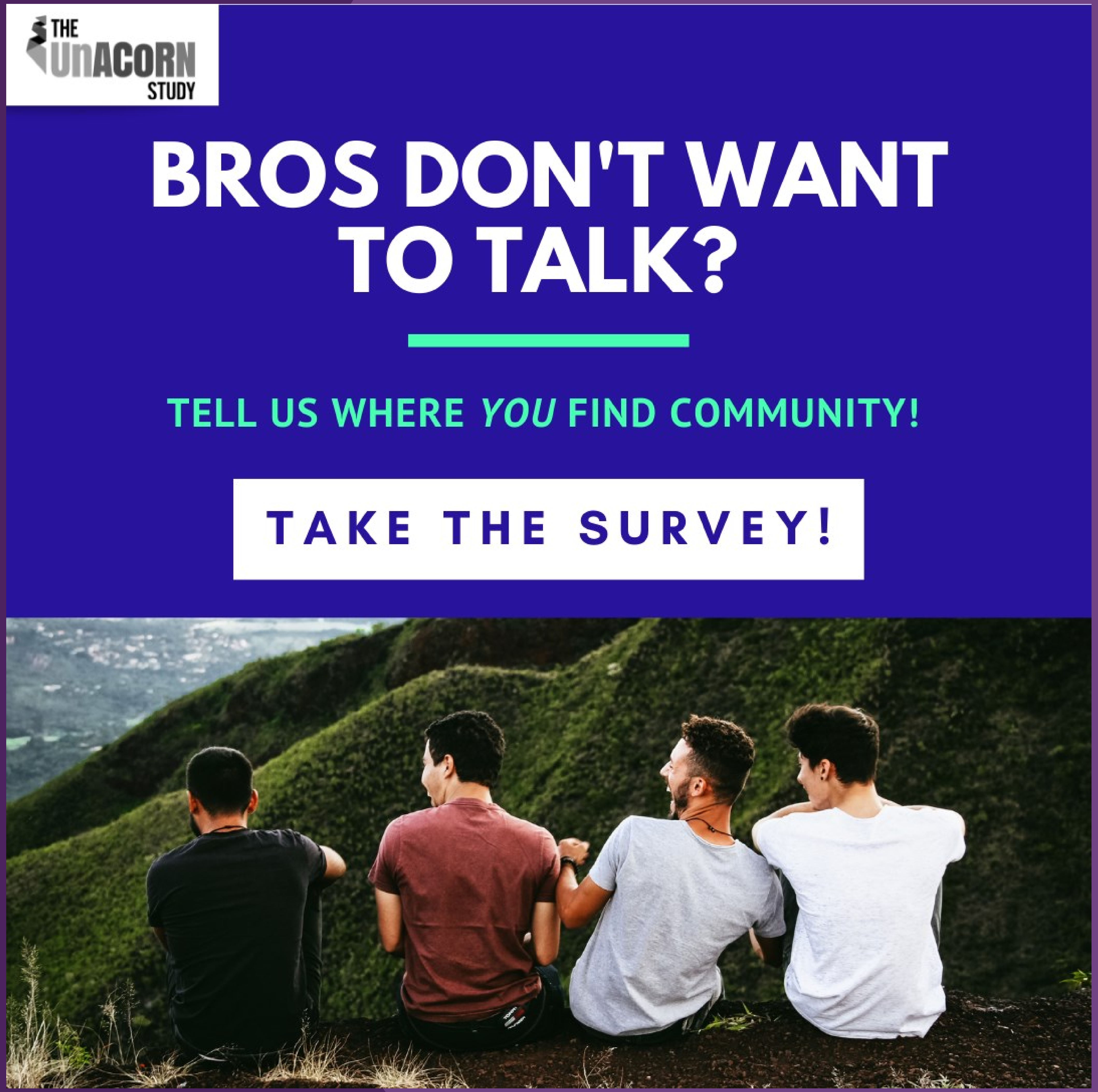

REAFFIRM COLLABORATIVE

Key takeaways

- 2S/LGBTQ-friendly framing works for sexual and gender minorities.
- Cis-het participants might have felt out of place, although they were eligible as study participants.
- Online campaigns skew towards what is most effective, creating recruitment “gaps.”
- Diversifying your campaign can mitigate these effects.

REAFFIRM COLLABORATIVE

Learn more:

unacorn.ca

REAFFIRM COLLABORATIVE
